# Relationship between gut microbiota and osteoarthritis: a scientometric analysis

**DOI:** 10.3389/fmicb.2025.1608800

**Published:** 2025-09-03

**Authors:** Zhicheng He, Songrui Xu, Ningning Ma, Yin Zuo, Xinyu Chen, Ting Yan, Pengcui Li, Yongchun Pan, Xiaochun Wei, Zhi Tian

**Affiliations:** ^1^Second Clinical Medical College, Shanxi Medical University, Taiyuan, Shanxi, China; ^2^Department of Orthopedics, The Second Hospital of Shanxi Medical University, Shanxi Key Laboratory of Bone and Soft Tissue Injury Repair, Taiyuan, Shanxi, China; ^3^Translational Medicine Center, Shanxi Medical University, Taiyuan, Shanxi, China; ^4^Department of Orthopedics, Third People’s Hospital of Datong City, Datong, Shanxi, China; ^5^Department of Orthopedics, Changzhi Yunfeng Hospital, Luzhou/Changzhi, Shanxi, China; ^6^Research Center for Reverse Microbial Etiology, Workstation of Academician, Shanxi Medical University, Taiyuan, China

**Keywords:** osteoarthritis, gut microbiota, bibliometrics, visualization, CiteSpace

## Abstract

**Objective:**

This study aims to visualize and analyze the literature on osteoarthritis (OA) and gut microbiota (GM) over the past decade (2011–2024) using bibliometric methods, and to understand the current research status and development trend in this field.

**Methods:**

Web of Science Core Collection was used as the source of literature, and the time limit was from January 2011 to July 2024, and the topics included “osteoarthritis” and “gut microbiota.” The included literature was analyzed in terms of annual distribution, country distribution, institutions, authors, keywords, and journals using Excel and CiteSpace.

**Results:**

A total of 192 articles were identified. Despite the limited volume, publication output demonstrated a consistent growth trend over the study period. Countries and institutions with the highest publication output and citation impact were predominantly located in China and the United States. The journals with the highest number of articles were mostly concentrated in Switzerland, and the research direction was mainly related to osteoarthritis and cartilage. The keywords appearing in the searched articles were gut microbiota, knee osteoarthritis, inflammation, rheumatoid arthritis, and gut microbiome.

**Conclusion:**

The research on OA and GM in China and abroad has shown an increasing trend, and the content of the research has been deepening with the passage of time. While the precise mechanisms remain elusive, targeting GM modulation emerges as a promising therapeutic strategy for OA, with potential clinical applications in disease prevention and management.

## 1 Introduction

Osteoarthritis (OA) is the most common degenerative disease of the joints, affecting one or more joints. It can occur in the hands, small joints of the spine, hips, knees, and feet. When it occurs in any joint due to trauma, sepsis, surgery, or metabolic injury, it is referred to as secondary osteoarthritis (Martel-Pelletier et al., 2016; Buchanan et al., 2023). A clinical diagnosis of OA can only be made when the patient is experiencing symptoms, and the goal of any intervention is to prevent or alleviate these symptoms (Glyn-Jones et al., 2015). Although the pathogenesis of OA remains largely unknown, exercise, diet, aging, obesity, strain, trauma, congenital anomalies of the joints, joint deformities, and many other factors have been recognized as potential risk factors for OA (Sun et al., 2019; Guss et al., 2019). In addition to being a major source of disability, OA reduces productivity and fitness on the individual level and increases health and welfare costs on the societal level (Tarride et al., 2012; Busija et al., 2013). The most important feature of OA is the irreversible nature of articular cartilage damage, so early treatment of OA and prevention of the disease are extremely important.

Notably, many of the aforementioned potential risk factors for OA are linked to the gut microbiota (GM), which is thus considered the primary causative factor for OA (Biver et al., 2019). Modulating GM may be a potential new intervention to address or prevent OA (Szychlinska et al., 2019). The GM in the human body is composed of more than 4,000 kinds of bacteria, as well as a variety of fungi, viruses, parasites, etc., that can synthesize a variety of vitamins and amino acids, participate in the metabolism of sugar and protein, and promote the absorption of mineral elements (Chen et al., 2023; Sommer and Bäckhed, 2013; Kundu et al., 2017; Almeida et al., 2021; Yan et al., 2021). Therefore, GM plays a vital role in maintaining various life activities as well as the dynamic balance of GM, which is highly susceptible to the influence of factors such as personal lifestyle, drugs (e.g., antibiotics), and dietary habits (Dixit et al., 2021). When the GM is out of dynamic balance, it can lead to the onset or development of OA (Wang et al., 2021). As research on OA intensifies, more and more researchers are finding that OA is closely related to GM.

Bibliometrics is the study of published papers, which involves the use of statistical data to analyze research trends and identify connections between different publications. Similar to epidemiology, researchers attempt to answer questions about a field based on data from related publications (Ninkov et al., 2021). Bibliometrics has become one of the common techniques for assessing the credibility, quality, and impact of scholarly work (Ellegaard and Wallin, 2015; Rondanelli et al., 2016). VOSviewer and CiteSpace are now common bibliometric visualization tools used for data analysis and visualization (Chen, 2004; van Eck and Waltman, 2010). The aim of this study is to use bibliometrics to analyze in-depth the literature in the field of OA and GM, and to show the current research status of the distribution trend and development of the relevant countries in this field through the CiteSpace software so as to provide scientific research direction and ideas for the research in this field.

## 2 Materials and methods

### 2.1 Data sources

**Figure 1:**
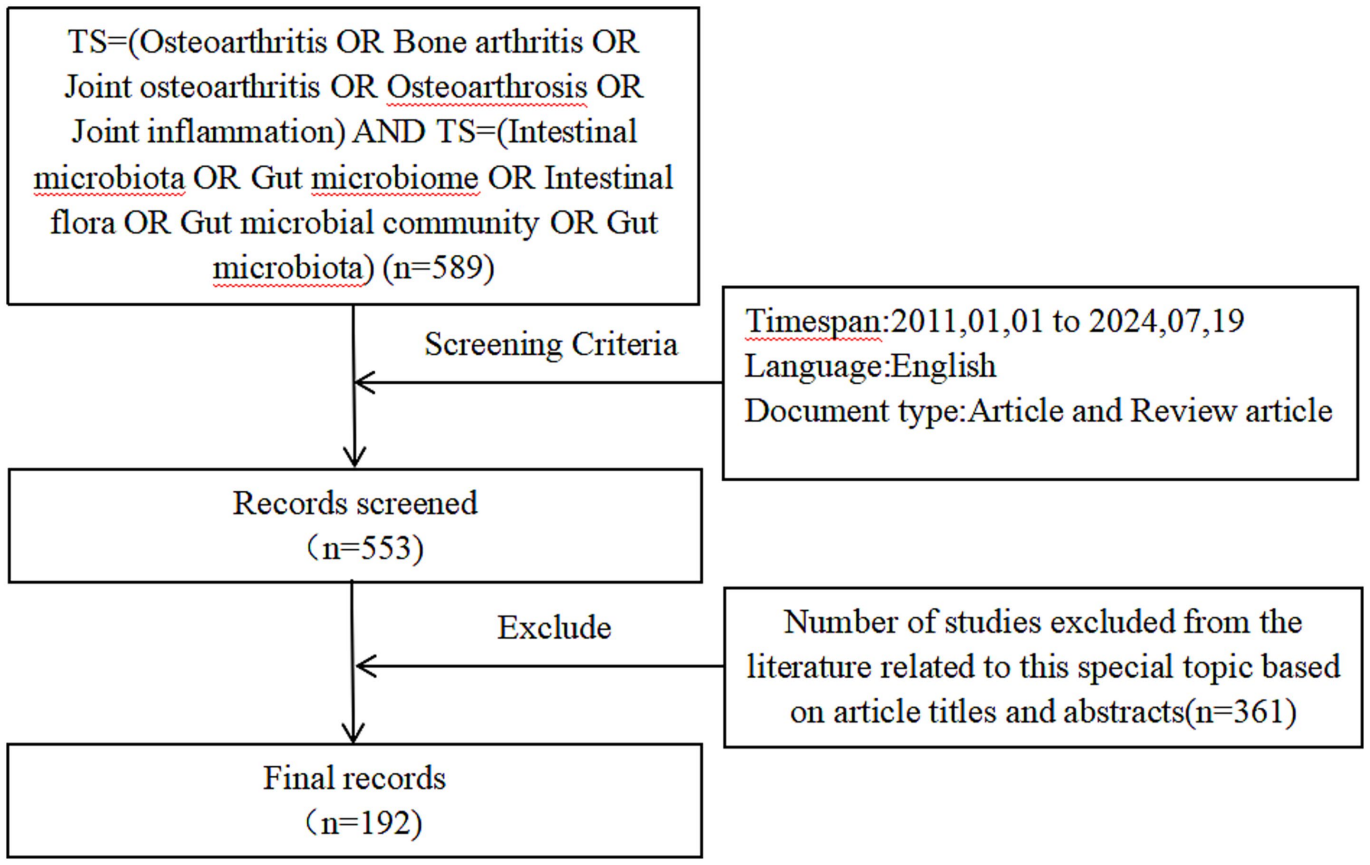
Flowchart for literature retrieval and screening.

Web of Science Core Collection is one of the largest and most comprehensive electronic scientific literature databases globally, renowned for its authoritativeness, reliability, rich bibliometric features, and wide application, which ensures the comprehensiveness, accuracy, and practicality of research data. Therefore, this study has selected the Web of Science Core Collection as the primary data source. The search was performed by using the Web of Science Core Collection for subject terms with the search strategy of (TS = (Osteoarthritis OR Bone arthritis OR Joint osteoarthritis OR Osteoarthrosis OR Joint inflammation)) AND TS = (Intestinal microbiota OR Gut microbiome OR Intestinal flora OR Gut microbial community OR Gut microbiota). The language was limited to “English,” and the type of literature was limited to “Article or Review Article.” A manual filtering process was employed to review all titles and abstracts of the relevant literature, excluding those unrelated to osteoarthritis, such as ankylosing spondylitis and rheumatoid arthritis, in order to enhance the accuracy and scientific rigor of the retrieved data, as these disease types fall outside the scope of this study. The literature search was completed on July 19, 2024, and 192 valid articles were included for analysis by deleting duplicates and filtering out articles that were irrelevant or had incomplete information. Figure 1 shows the process of article screening.

### 2.2 Data analysis

The screened 192 documents were imported into CiteSpace 6.3. R3 software and Microsoft Office Excel in full record format for visualization. We conducted a visual analysis using CiteSpace to detect contributions and collaborations among countries and institutions, citation and co-citation counts, research hotspots, and other potential information from a vast amount of literature. Specifically, by calculating the number of publications and citation frequencies each year, we measured research productivity and impact. And Microsoft Office Excel was used to perform journal analysis. The results of these performance analyses provided us with valuable insights into the development trends of OA and GM.

## 3 Result

### 3.1 Trend analysis of the volume of publications

**Figure 2:**
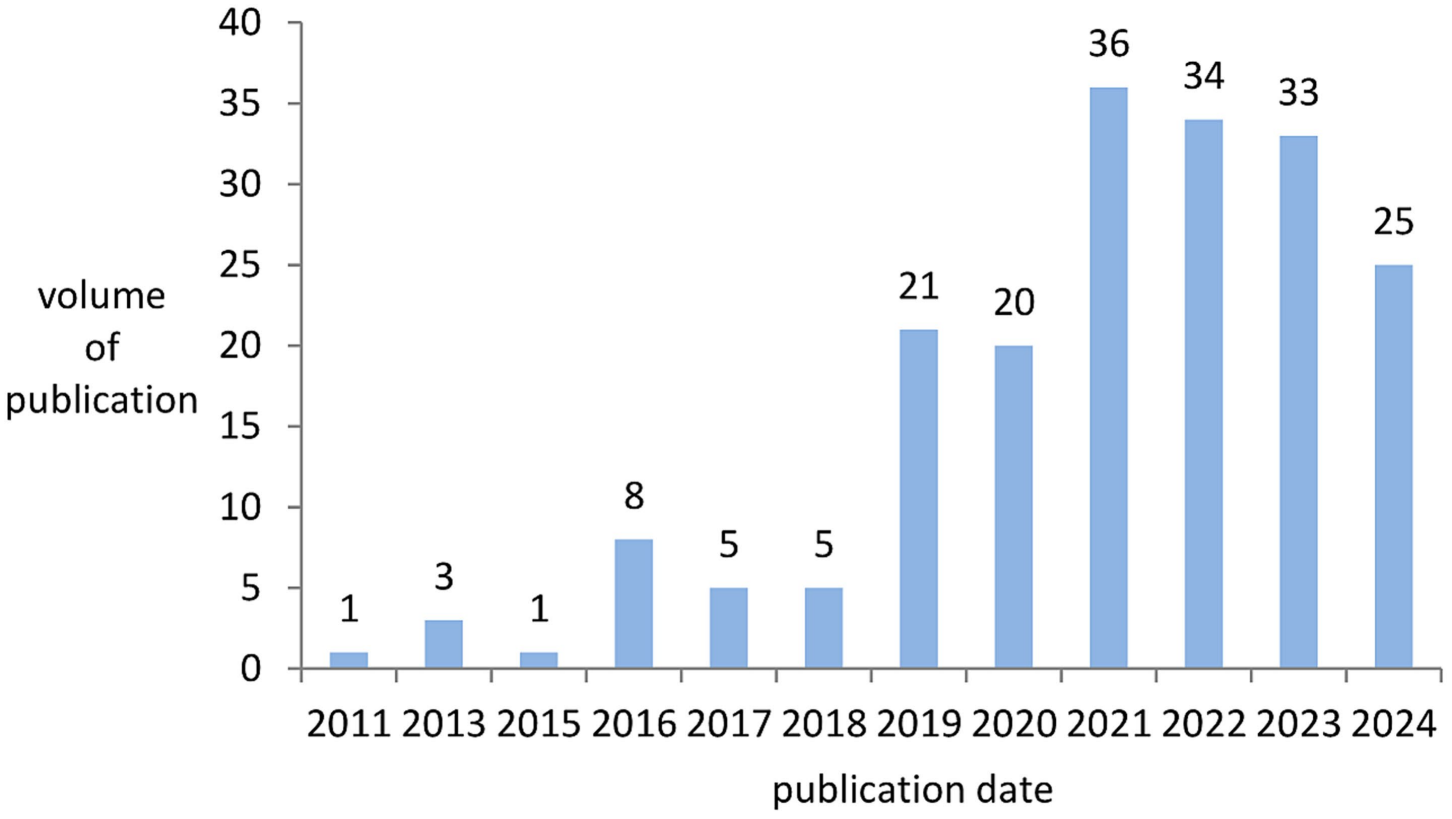
Bar chart of annual publications from 2011 to 2024.

A total of 192 papers, published between 2011 and July 19, 2024, in the research field related to OA and GM, were retrieved from the Web of Science. Overall, the number of publications indicates a trend of increase. The average annual number of publications from 2011 to 2018 was fewer than ten, and the number of publications in 2019 began to exhibit a clear upward trend, reaching a peak of 36 publications in 2021. Among them, the highest annual growth rate of publications in 2016 was 700.00%, followed by 2019 with an annual growth rate of publications at 320.00%. The average annual compound growth rate of publications from 2011 to 2024 was 28.10%. As presented in Figure 2, the annual increase in the number of articles published year by year shows that OA and GM is an active research field and has attracted wide attention from scholars.

### 3.2 Analysis of national co-occurrence networks

**Figure 3:**
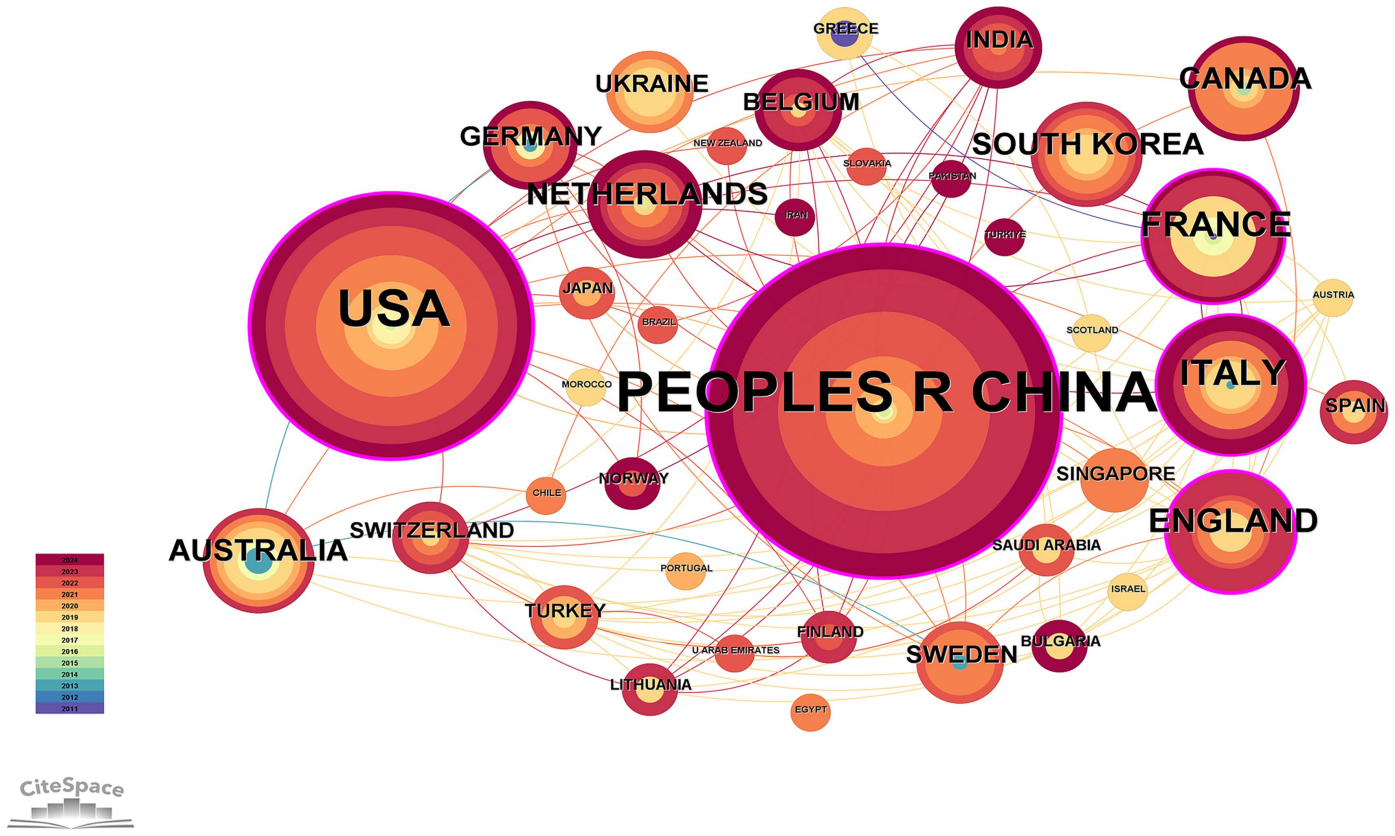
Network diagram of national cooperation in the field of osteoarthritis and gut microbiota. The size of each node represents the total number of publications from a given country, with larger nodes indicating higher research output. Node color reflects the year of publication, where warmer colors indicate more recent publications. The thickness of the connecting lines indicates the strength of the co-authorship relationship, measured by the frequency of joint publications between two countries.

Figure 3 illustrates that a total of 39 countries worldwide participated in the study of OA and GM. The data statistics reveal that, as shown in Table 1, China published the highest number of related literature with 79 articles, constituting 41.15%, followed by the USA with 51 articles (26.56%), Italy with 14 articles (7.29%), France with 13 articles (6.77%), and England with 11 articles (5.73%).

**Table 1:** Top 10 productive countries.

Rank	Country	Record Count	Frequency of citations for each article
1	China	79	41.15
2	USA	51	55.43
3	Italy	14	7.29
4	France	13	6.77
5	England	11	5.73
6	Netherlands	9	4.69
7	Canada	8	4.17
8	South Korea	8	4.17
9	Australia	8	4.17
10	Germany	6	3.13

### 3.3 Analysis of institution

**Figure 4:**
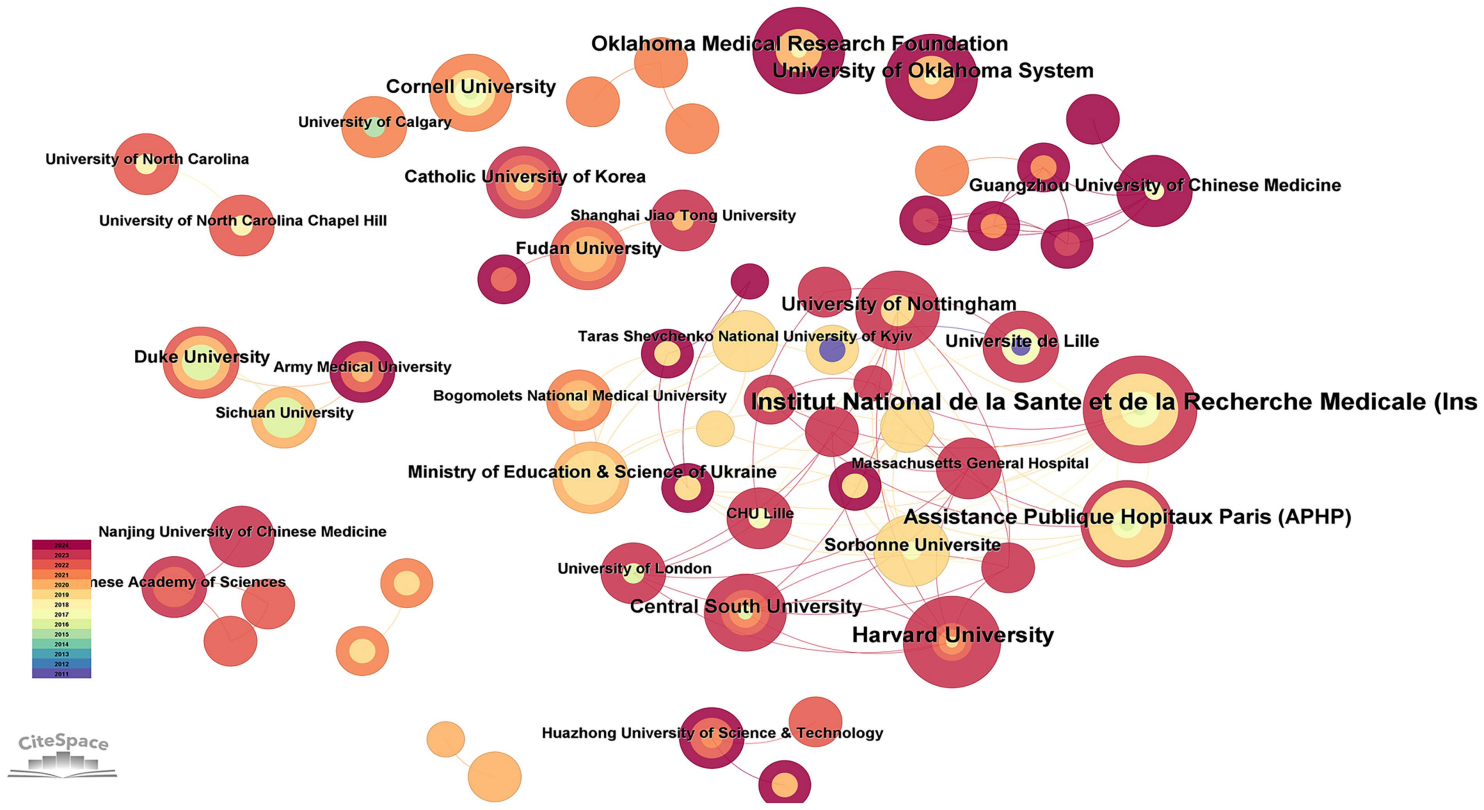
Institutional cooperation in the field of osteoarthritis and gut microbiota. The size of the nodes is determined by the number of papers. The color indicates the year of publication and the thickness of the line indicates the strength of the co-authorship relationship.

Figure 4 indicates that 171 institutions have conducted research in the field related to OA and GM. As we can see in Table 2, the Institut National de la Sante et de la Recherche Medicale (Inserm) contributed 9 articles (4.69%), Harvard University 7 articles (3.65%), Assistance Publique Hopitaux Paris (APHP) 6 articles (3.13%), the Oklahoma Medical Research Foundation 6 articles (3.13%), and the University of Oklahoma System 6 articles (3.13%).

**Table 2:** Top 10 productive institutions.

Rank	Institution	Record count
1	Institut National de la Sante et de la Recherche Medicate(Inserm)	9
2	Harvard University	7
3	Assistance Publique Hopitaux Paris (APHP)	6
4	Oklahoma Medical Research Foundation	6
5	University of Oklahoma System	6
6	Central South University	5
7	University of Nottingham	5
8	Cornell University	5
9	Duke University	4
10	Fudan University	4

### 3.4 Analysis of author

**Figure 5:**
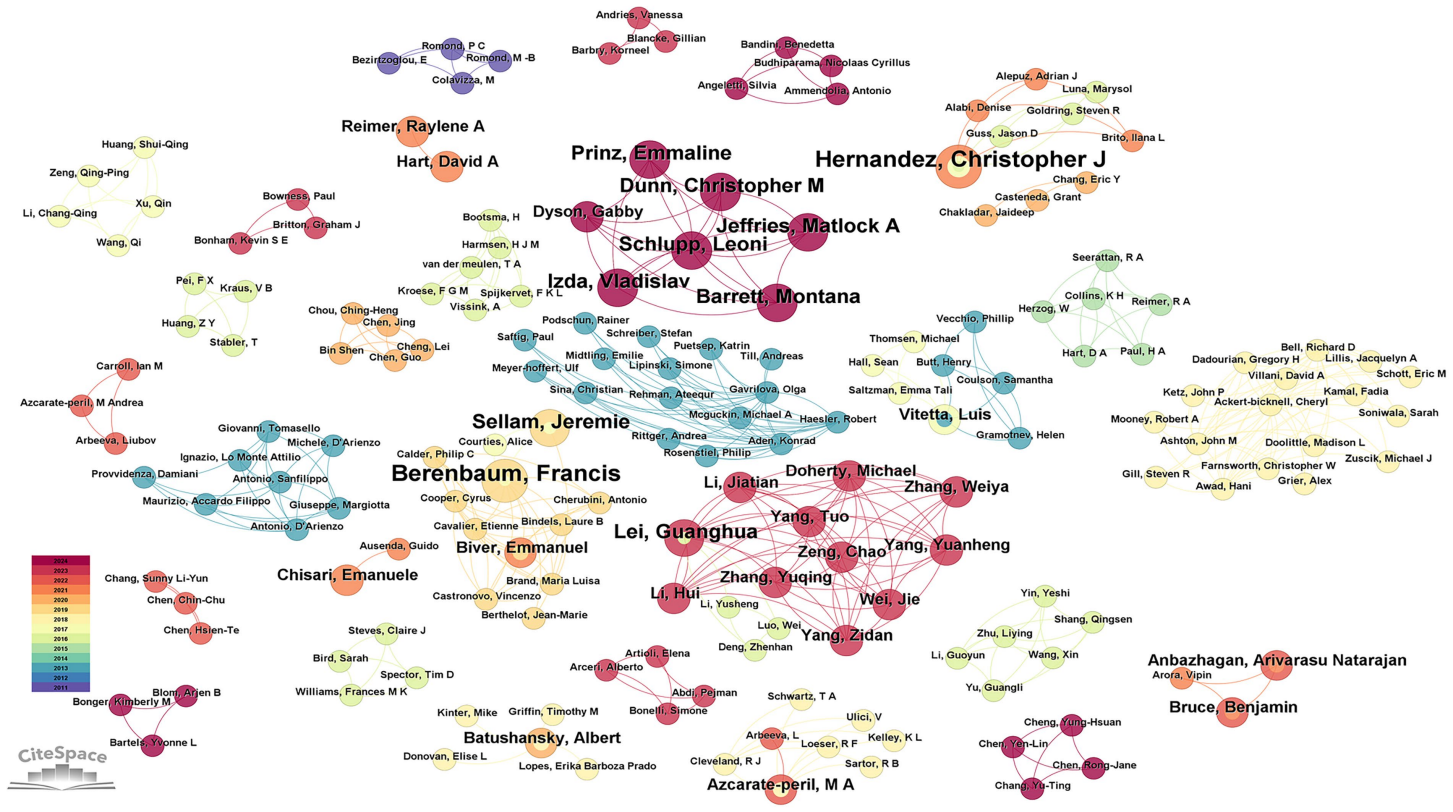
Network diagram between authors in the field of osteoarthritis and gut microbiota. The size of the nodes is determined by the number of papers. The color indicates the year of publication and the thickness of the line indicates the strength of the co-authorship relationship.

By analyzing the number of papers published by authors and the collaboration network through the 192 articles retrieved by CiteSpace, we identified 280 authors in the field of studying OA and GM. The current experts conducting research in this related field are Hernandez, Christopher J (4 articles, 2.08%), Berenbaum, Francis (4 articles, 2.08%), Lei, Guanghua (3 articles, 1.56%), Chen, Yu (3 articles, 1.56%), Sellam, Jeremie (3 articles, 1.56%), and Schlupp, Leoni (3 articles, 1.56%) (Table 3). By analyzing the cooperation between them, we can see that the cooperation between the scholars is distributed in a decentralized way. For example, Schlupp, Leoni, Dunn, Christopher M, Prinz, Emmaline, Izda, Vladislav, and Jeffries, Matlock A five scholars have a closer cooperation between them. Berenbaum, Francis and Sellam, Jeremie from Hôpital Saint Antoine, Assistance Publique - Hôpitaux de Paris established a close cooperation. As shown in Figure 5, on the visualization of author collaboration.

**Table 3:** Top 10 productive authors.

Rank	Author	Record count
1	Hernandez, Christopher J	4
2	Berenbaum, Francis	4
3	Lei, Guanghua	3
4	Chen, Yu	3
5	Sellam, Jeremie	3
6	Schlupp, Leoni	3
7	Dunn, Christopher M	3
8	Prinz, Emmaline	3
9	Izda, Vladislav	3
10	Jeffries, Matlock A	3

### 3.5 Analysis of keyword

**Figure 6:**
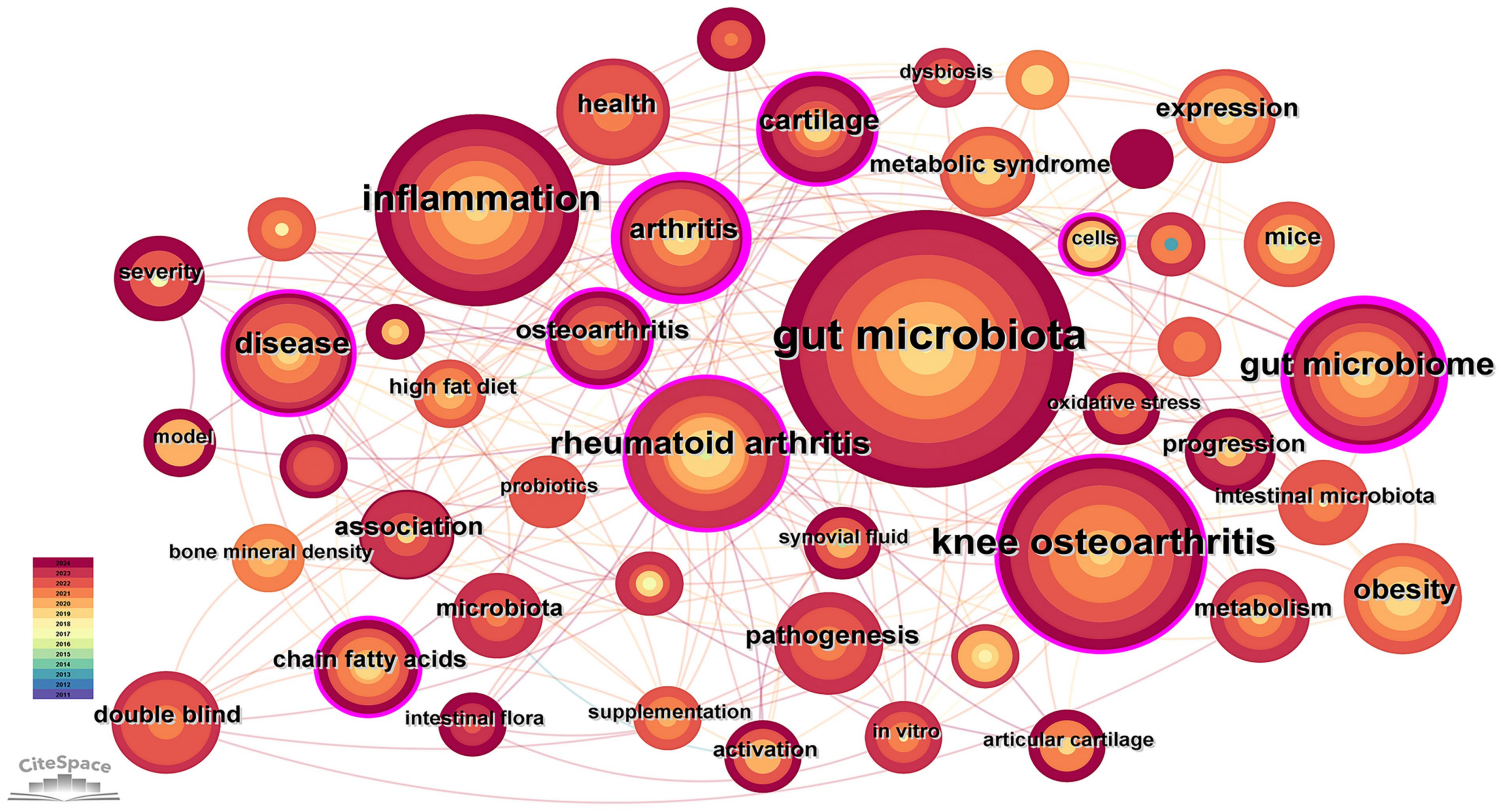
Co-occurrence keyword network in the field of osteoarthritis and gut microbiota. The size of the nodes is determined by the number of papers. The color indicates the year of publication and the thickness of the line indicates the strength of the co-authorship relationship.

**Figure 7:**
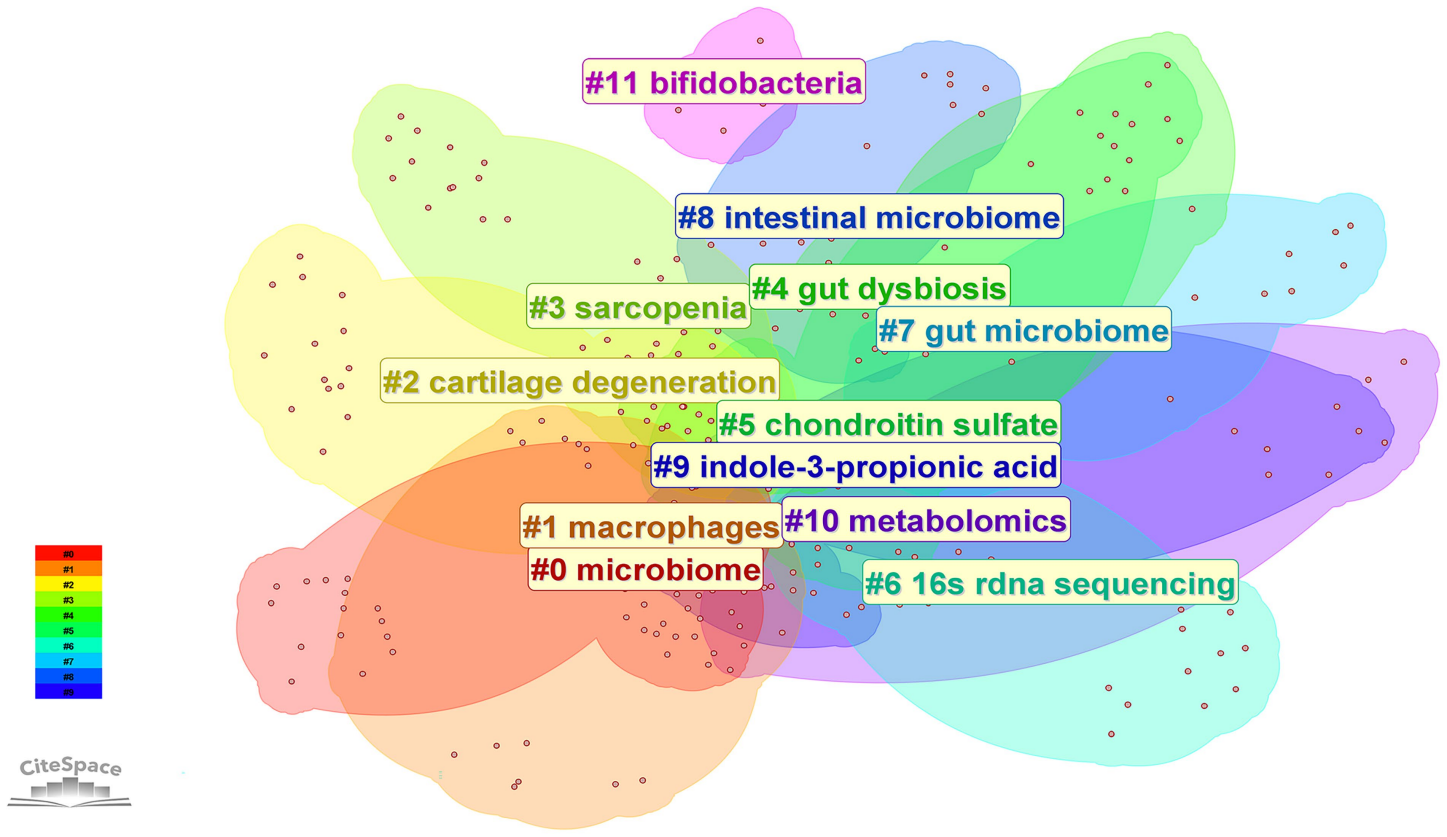
Clustered network of keyword analysis on osteoarthritis and gut microbiota. Keywords were clustered using CiteSpace based on their co-occurrence frequency and association strength. Different colors represent distinct clusters, and each cluster is labeled with its representative term.

The 192 retrieved articles were analyzed for keywords using CiteSpace. Keywords are a highly refined version of the content of an article and can highlight the main content of the article. As indicated by Figure 6, a total of 285 keywords were analyzed within this field, and the top 10 keywords in terms of frequency of occurrence are presented in Table 4. Through keyword clustering analysis, the current research direction in this field can be clarified. As shown in Figure 7, the research revolves around the keyword GM, which can be classified into twelve clusters according to the keyword color, which are #0 microbiome, #1 macrophages, #2 cartilage degeneration, #3 sarcopenia, #4 gut dysbiosis, #5 chondroitin sulfate, #6 16 s rdna sequencing, #7 gut microbiome, #8 intestinal microbiome, #9 indole-3-propionic acid, #10 metabolomics, and #11 bifidobacteria.

**Table 4:** Top 10 productive keywords.

Rank	Keyword	Frequency
1	gut microbiota	100
2	knee osteoarthritis	54
3	inflammation	53
4	rheumatoid arthritis	27
5	gut microbiome	27
6	disease	23
7	arthritis	19
8	obesity	17
9	cartilage	16
10	health	15

**Figure 8:**
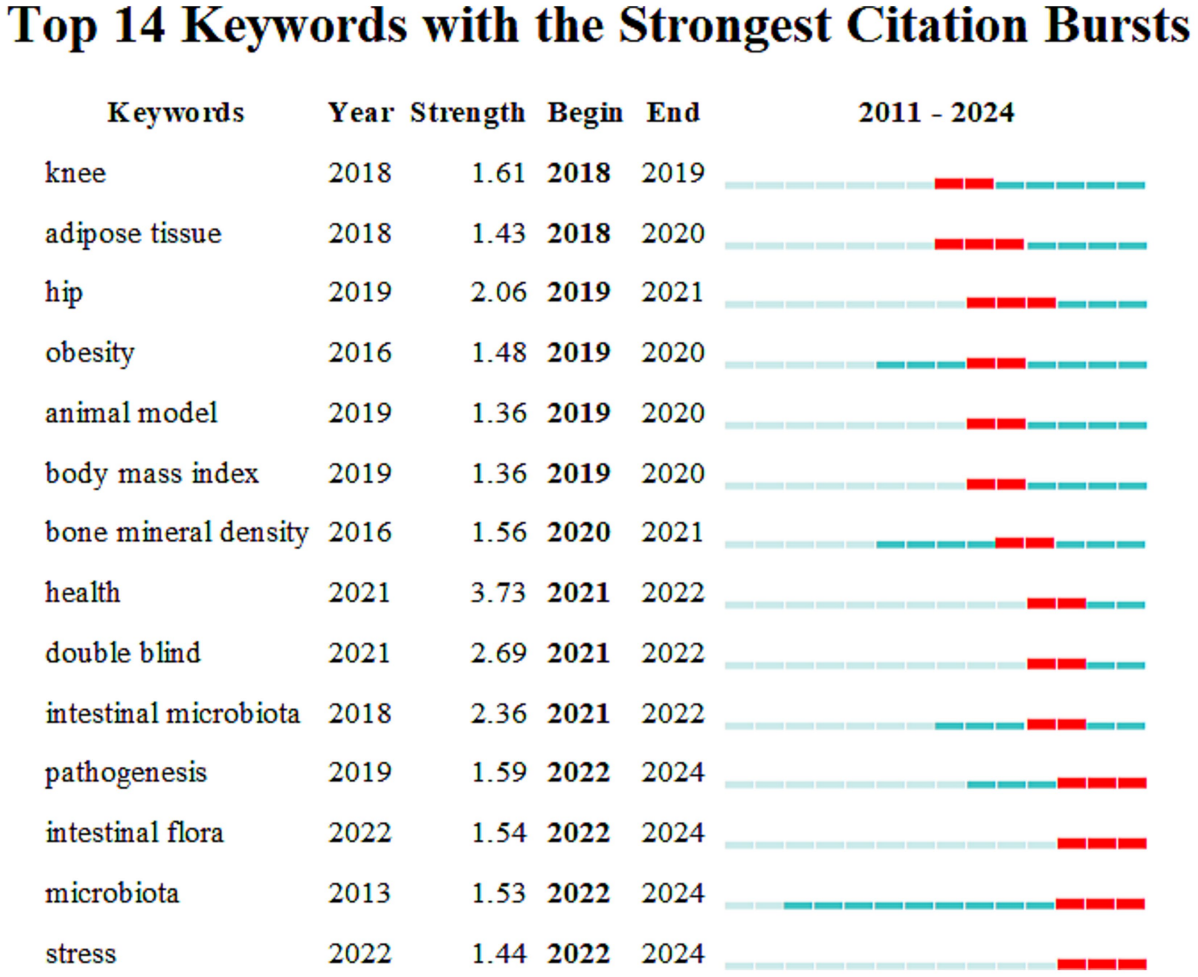
Top 14 keywords with the strongest citation bursts. The vertical axis lists the top 14 keywords ranked by burst strength, while the horizontal axis represents the time span from 2011 to 2024. Each horizontal bar corresponds to a keyword, with the green segment showing the overall time range of the publications containing that keyword, and the red segment indicating the burst period when its citations increased sharply.

The so-called “burst words” refer to words that are frequently quoted within a period. CiteSpace is used to detect burst keywords, which are considered indicators of research frontier topics. The timeline is represented by a blue line, and the time interval of keyword bursts is represented by the red part on the blue timeline. It represents the start year, end year, and duration of the burst. Figure 8 indicates that the top 14 burst words, ranked by their burst time, are as follows: knee (2018–2019), adipose tissue (2018–2020), hip (2019–2021), obesity (2019–2020), animal model (2019–2020), body mass index (2019–2020), bone mineral density (2020–2021), health (2021–2022), double blind (2021–2022), intestinal microbiota (2021–2022), pathogenesis (2022–2024), intestinal flora (2022–2024), microbiota (2022–2024), and stress (2022–2024).

### 3.6 Analysis of journal

Journal analysis of the 192 retrieved articles was performed by Excel software, and Table 5 presents the top 10 journals in terms of the number of OA and GM -related studies published from 2011 to July 2024. Of these, there were five journals from Switzerland, with the remaining two journals from the United States, two from England, and one from France.

**Table 5:** Top 10 productive journals.

Rank	Journal	Country	Record count	IF (2023)
1	Osteoarthritis and Cartilage	England	11	7.2
2	Nutrients	Switzerland	10	4.8
3	International Journal of Molecular Sciences	United States	8	4.9
4	Frontiers in Microbiology	Switzerland	7	4
5	Arthritis & Rheumatology	United States	5	11.4
6	Frontiers in Immunology	Switzerland	5	5.7
7	Biomedicines	Switzerland	4	3.9
8	Frontiers in Cellular and Infection Microbiology	Switzerland	4	4.6
9	Joint Bone Spine	France	4	3.8
10	Arthritis Research & Therapy	England	3	4.40

### 3.7 Analysis of cited references

The articles were analyzed for citations, and the authors of the top 10 cited articles (Table 6) were, in order: Collins et al. (2015) (cited 167 times); Boer et al. (2019) (cited 163 times); Huang and Kraus (2016) (cited 158 times); Schott et al. (2018) (cited 156 times); Huang et al. (2016) (cited 150 times); Courties et al. (2017) (cited 145 times); Hernandez et al. (2016) (cited 138 times); Biver et al. (2019) (cited 89 times); Rios et al. (2019) (cited 85 times); and van der Meulen et al. (2016) (cited 84 times).

**Table 6:** Top 10 cited articles.

First author	Count	Year	Literature	Journal
Kelsey H Collins	167	2015	Relationship between inflammation, the gut microbiota, and metabolic osteoarthritis development: studies in a rat model	Osteoarthritis and Cartilage
Cindy G Boer	163	2019	Intestinal microbiome composition and its relation to joint pain and inflammation	Nature Communications
Zeyu Huang	158	2015	Does lipopolysaccharide-mediated inflammation have a role in OA?	Nature Reviews Rheumatology
Eric M Schott	156	2018	Targeting the gut microbiome to treat the osteoarthritis of obesity	JCI Insight
Z Y Huang	150	2016	Both systemic and local lipopolysaccharide (LPS) burden are associated with knee OA severity and inflammation	Osteoarthritis and Cartilage
Courties A	145	2017	Metabolic syndrome-associated osteoarthritis	Current Opinion in Rheumatology
Christopher J Hernandez	138	2016	Links Between the Microbiome and Bone	Journal of Bone and Mineral Research
Emmanuel Biver	89	2019	Gut microbiota and osteoarthritis management: An expert consensus of the European society for clinical and economic aspects of osteoporosis, osteoarthritis and musculoskeletal diseases (ESCEO)	Ageing Research Reviews
Jaqueline Lourdes Rios	85	2019	Protective effect of prebiotic and exercise intervention on knee health in a rat model of dietinduced obesity	Scientific Reports
T A van der Meulen	84	2016	The microbiome-systemic diseases connection	Oral Diseases

## 4 Discussions

The main objective of this study was to assess the hotspots and trends of research related to OA and GM through bibliometric analysis. The analysis showed that over the past decade or so, the number of studies related to OA and GM has increased year by year. The Web of Science core database showed that the number of publications was 1 in 2011 and 33 in 2023, which is a 33-fold increase, and as many as 36 in 2021, reflecting the growing interest of researchers in the relationship between OA and GM. The potential reason may be that with the advancement of science and technology and the deepening of medical research, an increasing number of clinical studies and observational data have shown that changes in GM may be related to the occurrence, development, and severity of symptoms in OA (Jiang and Shen, 2023; Jeyaraman et al., 2023; Chen et al., 2022). Consequently, research institutions have continuously increased their investment in this research field, providing important material support for studies on OA and GM. And promoting the initiation of research projects and the output of research results. By statistically analyzing publications by country, institution, and journal, we identified the key contributors and collaborative patterns in the OA and GM field. China, the United States, and Italy emerged as the leading countries, indicating that China and the United States hold a particularly prominent position. Several factors may explain their dominance. In China, rapid growth in translational medicine programs, substantial national funding for microbiome-related projects, and an aging population with a rising OA burden have driven output. In the United States, sustained support from the National Institutes of Health (NIH) and other federal agencies, together with well-established microbiome research infrastructure, has facilitated both basic and clinical studies. In addition, France, England, the Netherlands, and other countries have also developed close cooperation. Such cross-national collaborations may accelerate knowledge transfer, harmonization of methodologies, and the implementation of multicenter clinical and preclinical studies, thereby advancing the translational potential of OA and GM research. Among the top 10 institutions, five are from the United States, with the remaining two institutions from China, two from France, and one from England, with the Institut National de la Sante et de la Recherche Medicate (Inserm) being the institution with the highest number of relevant publications. Institut National de la Sante et de la Recherche Medicate (Inserm) has contributed more articles in this field, which explores the pathogenesis of OA from the perspective of metabolic syndrome as well as discusses the relationship between OA and intestinal flora (Biver et al., 2019; Courties et al., 2017; Speca and Dubuquoy, 2017; Courties et al., 2019; Piva et al., 2023; Berthelot et al., 2019), and also provides insights into the mechanisms of increased intestinal permeability and intestinal inflammation in the pre-pathogenesis of arthritis, which provides new ideas and approaches for the prevention and treatment of arthritis (Hecquet et al., 2023). Six of the top ten authors are from the United States, five of whom are from the Oklahoma Medical Research Foundation. The United States has a strong overall strength in biomedical research, with abundant scientific research resources, advanced equipment, and rich experience. Furthermore, American research institutions have made significant investments in the fields of OA and GM research, attracting a large number of outstanding scientific talents. This has led to the formation of a relatively comprehensive research system and a collaborative network, thereby driving the in-depth development of research in these areas. These factors, working together, have made the United States prominent in research outcomes within these fields, resulting in a concentration of related research among American authors. Hernandez, Christopher J has contributed the most articles in the field. Hernandez, Christopher J focuses on the link between the microbiome and bone health (Luna et al., 2020; Hernandez, 2017; Hernandez, 2020), which has also been demonstrated through animal studies that have shown that gut microbes mediate the effects of obesity on cartilage degeneration, suggesting a potential role for the gut microbiome in the pathogenesis of OA (Hernandez, 2017). The keywords appearing more frequently in the searched literature are GM, knee osteoarthritis, inflammation, rheumatoid arthritis, and gut microbiome. Among the top 10 journals, there are five journals from Switzerland, the remaining two are from the United States, and two are from England.

The bibliometric analysis revealed several high-frequency keywords and clusters—such as “gut microbiota”, “knee osteoarthritis”, “inflammation”, “cartilage degeneration, “and “gut dysbiosis”—that map onto emerging mechanistic insights into GM–OA interactions. For the mechanism of the role of GM in the pathogenesis of OA, it has been shown that the integrity of the intestinal barrier contributes to delaying the progression of OA, and by fully understanding the mechanism by which intestinal flora affects the intestinal barrier, it may be helpful to further understand the link between OA and GM, and then to improve the OA through precise interventions (Wei et al., 2022). Elevated relative and absolute abundance of Streptococcus spp. in the gut microbiota was significantly and positively correlated with the degree of knee pain associated with OA. The underlying mechanisms may be due to an increase in metabolites passing through the intestinal blood barrier on the one hand, but also through the activation of immunogenic products in local or systemic macrophages, triggering an inflammatory cascade response (Boer et al., 2019). Several animal studies have shown that obesity, high-fat, and high-sugar diets all lead to a state of dysbiosis in the GM and that these changes are associated with elevated serum levels of lipopolysaccharides, which leads to OA (Carbonero et al., 2021). It has also been shown that depletion of the GM has an impact on the development of OA (Guan et al., 2022). Studies have also summarized the research evidence supporting the “gut-joint axis” hypothesis and the interactions between the GM and factors associated with OA, elucidating the underlying mechanisms of this complex interaction (Hao et al., 2021; Guido et al., 2021). Berthelot et al. (2019) also found that DNA from the GM can be found in synovial membranes of OA and in human cartilage. Thus, a variety of intestinal flora plays a crucial role in the development of OA (Yu et al., 2021).

The focus of future research in this area should be on how various dietary supplements can regulate the GM to improve or prevent the development of OA. Some studies have demonstrated that the addition of probiotics and other substances can increase beneficial intestinal bacteria and thus improve intestinal health, which lays the foundation for the development of research related to the regulation of GM by dietary supplements (Arora et al., 2021; Favazzo et al., 2020; Liu et al., 2019). A study by Schott et al. (2018) demonstrated that intervention with a specific indigestible prebiotic fiber, oligofructose, was effective in restoring a healthy gut microbial community, significantly reducing knee inflammation and protecting cartilage, and ultimately preventing the development of OA in an obesity-induced mouse model of OA. In addition, based on the high-impact studies identified by our bibliometric analysis, lipopolysaccharide (LPS) is consistently recognized as a key molecular bridge connecting gut ecological dysregulation, systemic inflammation, and OA pathogenesis. These studies suggest the potential therapeutic value of targeted reduction of LPS levels (e.g., through lifestyle interventions such as high-fiber diets and exercise, or medical treatments such as microbiome transplantation) (Collins et al., 2015; Huang and Kraus, 2016; Huang et al., 2016). The effective utilization of GM and their metabolites remains relatively uncommon, suggesting that it could be a potential therapeutic avenue for preventing and intervening in the development of OA (Liu et al., 2022). Sun et al. (2023) also suggested that future studies should include more new interventions on immune cell modifications and gene regulation of specific GM associated with OA in order to validate the application of GM regulation in the pathogenesis of OA. Therefore, hereafter researchers should give more consideration and close attention to the GM of OA patients, not only focusing on the bone and joint and its cartilage (Ramires et al., 2022). Researchers can achieve innovative microbe-targeted therapies by developing protocols that predict inflammatory joint disease flare-ups based on gut ecological dysregulation (Longo et al., 2024).

## 5 Limitations

This study has certain limitations. First, we relied solely on the Web of Science Core Collection database for literature retrieval. While Web of Science Core Collection database covers a wide range of high-impact journals, relevant studies indexed in other major databases such as PubMed, Embase, or Scopus, as well as those published in non-English journals, may have been overlooked. This reliance on a single data source could lead to the omission of region-specific research findings and non-English-language studies, thereby limiting the diversity of perspectives. Future studies should address this limitation by integrating multiple databases and adopting multilingual search strategies to enhance the comprehensiveness and representativeness of findings. Second, bibliometric analyses inherently focus on quantitative metrics such as the number of publications, citation frequency, and keyword co-occurrence without directly assessing the quality, mechanistic depth, or clinical translational value of individual studies; and keyword networks, although reflective of hotspots, are difficult to distinguish disease-specific mechanisms. Finally, to ensure that studies were relevant to osteoarthritis, we performed manual screening to exclude studies that focused primarily on other rheumatic diseases (e.g., rheumatoid arthritis, ankylosing spondylitis, and gout). This included reviewing article titles, abstracts, and, where necessary, reading the full text to confirm that the primary research focus met our inclusion criteria. However, some cross-disease literature may still appear in the network due to terminological overlap and the presence of concurrent diseases, which may affect the interpretation of certain keyword clusters. Despite these constraints, the general trends remain within an acceptable range.

## 6 Conclusion

This study analyzes the current hotspots and trends of research in the field of OA and GM based on bibliometric studies to provide a certain reference basis for domestic and international research in this field. Overall, the relevant studies on OA and the GM have been increasing annually, and the mechanism of the GM on OA has been studied more deeply. In the future, greater attention should be given to preventing and treating OA through dietary regulation of intestinal flora and GM transplantation, among other intervention strategies. Additionally, the pathogenesis of OA can be investigated by integrating multi-omics research approaches, such as metabolomics and transcriptomics, with insights from GM and OA studies. This study may provide valuable information for future researchers and accelerate the development of this field. Future efforts should focus on collecting additional data to investigate the potential mechanisms through which GM influences OA.

## Data Availability

The original contributions presented in the study are included in the article/supplementary material, further inquiries can be directed to the corresponding authors.
